# Appraisal of Periodontal Condition amongst Leather Manufacturing Plant Workers in Central India: A Prevalence Survey

**DOI:** 10.1155/2019/6037929

**Published:** 2019-09-22

**Authors:** Ashwini Dayma, P. Amith, Venkat Raman Singh, Nilotpol Kashyap

**Affiliations:** ^1^Department of Community & Preventive Dentistry, UCMS College of Dental Surgery, Siddharthanagar, Nepal; ^2^Department of Periodontology, UCMS College of Dental Surgery, Siddharthanagar, Nepal; ^3^Department of Pedodontics & Preventive Dentistry, UCMS College of Dental Surgery, Siddharthanagar, Nepal

## Abstract

**Objective:**

To assess the periodontal status among the leather factory workers in Dewas and to provide a baseline data for the oral health care and promotion programs.

**Materials and Methods:**

A total of 850 study population was considered for examination, and the age ranged from 20 to 60 years. All those factory workers with low socioeconomic status and poor background were considered. The data were collected by means modified World Health Organization (WHO) Oral Health Assessment 1997 Performa and clinical examination with the use of the Community Periodontal Index (CPI). Statistical analyses were used: chi-squared test, Mann–Whitney *U* test, and Kruskal–Wallis test.

**Results:**

The sociodemographic characteristics were found to be significantly associated with the periodontal status in the study population (*p* ≤ 0.05). Mean number of loss of attachment (LOA) 0 sextants was 3.55 ± 2.35, LOA 1 was 0.935 ± 1.3, LOA 2 was 0.414 ± 0.8, LOA 3 was 0.529 ± 0.94, and LOA 4 was 1.24 ± 0.04; all the parameters showed statistically significant difference (*p*=0.001) except LOA 1 and LOA 2.

**Conclusion:**

The periodontal health status of the factory workers is poor. Factors associated with high prevalence of periodontal disease could be the following: a high rate of tobacco consumption and not cleaning their teeth at all along with other associated factors like stress, poor oral hygiene, etc.

## 1. Introduction

The leather industry holds a prominent place in the Indian economy. India is the second largest producer of footwear and leather garments in the world. The leather industry is an employment-intensive sector, providing jobs to about 2.5 million people, mostly from the weaker sections of the society. Women employment is predominant in leather products sector with about 30% share [1]. In the modern era, the growth of a nation is measured by the amount of industrial progress. Such industrialization affects community health as well as worker's health. Occupational health problems are becoming more prevalent with rapid industrialization and mechanization in developing countries [2]. The major production centers for leather and leather products in India are located in the Madhya Pradesh (Dewas), Tamil Nadu, West Bengal, Uttar Pradesh, Maharashtra, Punjab, Karnataka, Andhra Pradesh, Rajasthan, Jammu and Kashmir, Haryana, Delhi, and Kerala [1]. In Madhya Pradesh, Dewas has many industries providing employment to thousands of industrial workers. Leather factory is one of largest factories among them, with different working plants which mainly include (1) tanning and (2) manufacturing (footwear) plants.

Health continues to be a neglected entity despite continuous efforts for health promotion, worldwide. Health is often taken for granted, and its value is not fully understood, until it is lost [2]. The occupational environment is one of the major determinants for health. Oral health is an integral part of general health and a valuable asset for any individual. According to the World Health Report 2003, the prevalence of periodontitis is 86% in India. Periodontal diseases are the major dental problems which affect people worldwide as well as the Indians [3]. Periodontal diseases are a group of chronic, progressive bacterial infections resulting in inflammation and destruction of tooth supporting tissues [4]. Its impact on individuals and communities in terms of pain and suffering, impairment of function, and reduced quality of life is considerable.

In this present period of evidence-based dentistry and oral health, foremost priority should be given to researches dealing with the prevalence of periodontal diseases. The literature on the periodontal health of leather workers is almost nonexistent. Therefore, the present study was conducted as an attempt to assess the periodontal health status in relation to place of work (plant), i.e., tanning and footwear.

## 2. Materials and Methods

A cross-sectional descriptive study was conducted among 900 leather factory workers in Dewas city in the state of Madhya Pradesh, India, to assess the periodontal status among the 20–60  years age group [5]. Dewas is situated in the Malwa plateau in west central part of Madhya Pradesh. The study protocol was reviewed by the Institutional Review Board, and it was granted ethical clearance.

Official permission was obtained from the industry authorities which were located in factory premises. To select these workers, a simple random technique was followed. Written informed consent was obtained from all the participants who fulfilled the eligibility criteria. The eligibility criteria are as follows: voluntary participation and workers who are not taking any medication, systemic healthy, and dentate.

### 2.1. Inclusion Criteria

- All permanent factory workers who have been working in the factory last 3 years
- All factory workers aged between 20–60 years

### 2.2. Exclusion Criteria

- Subjects not willing to participate in the study
- Subjects who are not present during the study period

The survey Performa consisted of two sections: (1) demographic data; (2) clinical parameters including Community Periodontal Index (CPI) and loss of attachment (LOA), collected by using the modified WHO Oral Health Assessment Form 1997. The CPI and LOA are dependent variables in the present study. The examination for the periodontal status was recorded by using the community periodontal index probe and a mouth mirror.

Before the commencement of the study, training and intraexaminer reproducibility were done in the Department of Public Health Dentistry. Forty subjects who possessed full range of periodontal condition examined, followed by their re-examination a week later which resulted in 87% of the diagnostic acceptability with *k* = 0.84. A pilot study was carried out among factory workers, to determine the feasibility of the study and also to assess the periodontal status. The examination was conducted from October 2015 to December 2015. Depending on the prevalence which was obtained (90%), a 95% confidence level, and a 5% allowable error, the determined sample size was 848 factory workers, which was rounded to 850. Based on the sample size calculation, anticipating noneligibility, and unwillingness to participate in the study, a sample of 900 individuals was chosen for this study out of 1457 factory workers.

The recorded data were compiled and entered into a spreadsheet computer program (Microsoft Excel 2007), and they were then exported to the data of SPSS, version 20 (SPSS Inc., Chicago, Illinois, USA). The descriptive statistics included the computation of the percentages, means, and standard deviations. The statistical tests which were applied for the analysis were Pearson's chi-squares test (*χ*^2^), Mann–Whitney *U* test, and Kruskal–Wallis test. For all the tests, the confidence interval and the *p* value were set at 95% and ≤0.05, respectively.

## 3. Result

### 3.1. Distribution of Periodontal Status

The age group, gender, and plant employment were recorded in this investigation. The overall prevalence of periodontal disease was 91.8% with incidence increasing with age. This relationship was found to be statistically highly significant. In all age groups, the majority of them had periodontal problems; in particular, there was not even a single person with healthy periodontium in the 35–44 and 45–54 years age group (Table 1).

Periodontal diseases were found more in tanning plant workers as compared to footwear plant workers. 137 (30.1%) tanning plant workers had shallow pocket as compared to 103 (26%) footwear plant workers while 132 (29%) tanning workers had deep pocket as compared to 42 (10.6%) footwear workers. Deep pockets were found more in male as compared to female. The periodontal status of factory workers shows statistically highly significant difference according to age groups, gender, and type of plant (*p*=0.001) (Table 2).

The overall mean number of the sextants for bleeding was 1.66 ± 0.05, while that for the calculus, shallow pockets (4-5 mm), and deep pockets (6 mm or more) was 1.28 ± 1.46, 1.4 ± 0.51, and 1.22 ± 0.08, respectively. The mean number of sextants affected by periodontal disease was increasing with age. Also the mean number of bleeding sextant was greater than other three periodontal indicators, i.e., 2.29 ± 1.76. The mean number of calculus sextant was 2.05 ± 2.11, while that of shallow pocket and deep pocket was 1.89 ± 1.8 and 2.37 ± 2.33, respectively. The parameter showed statistically highly significant difference according to age, gender, and type of plant (*p*=0.001) (Table 3).

### 3.2. Distribution of Loss of Attachment (LOA)

Among the 850 workers, loss of attachment 0 code was found among 351 (41.3%) workers: code 1 in 194 (22.8%), code 2 in 96 (11.3%), code 3 in 48 (5.6%), and code 4 in 161 (18.9%) workers. LOA was more in tanning plant workers as compared to footwear plant workers. 17 (3.7%) tanning plant workers had LOA 3 as compared to 31 (7.8%) footwear plant workers, while 86 (18.9%) tanning workers had LOA 4 as compared to 75 (18.9%) footwear workers. It was found more in male as compared to female. Loss of attachment of factory workers shows statistically highly significant difference according to age groups, gender, and type of plant (*p*=0.001) (Table 4).

The mean number of sextants affected by loss of attachment was increasing with the increase in age. The mean number of LOA 0 sextants was 3.55 ± 2.35, LOA 1 0.935 ± 1.3, LOA 2 0.414 ± 0.8, LOA 3 0.529 ± 0.94, and LOA 4 1.24 ± 0.04. All the parameters showed statistically significant difference according to age, gender, and type of plant except LOA 1 and LOA 2 (*p*=0.001) (Table 5).

## 4. Discussion

The present study was conducted to gather information on periodontal health of leather factory workers in Dewas city. In the study, it was found that a higher proportion of workers had periodontal diseases, with a prevalence of 91.8%. This finding was comparable to the finding of a study done by Amit et al. [6] who found the prevalence to be 92.75% among the same age group as the present study. Another study conducted by Umesh et al. [7] found CPI score for calculus 2.41 ± 0.49, shallow pocket 2.29 ± 0.83, and deep pocket 0.41 ± 0.02 in male and 2.06 ± 0.03 for calculus, 1.26 ± 0.23 for shallow pocket, and 0.32 ± 0.04 for deep pocket in female, respectively, which were less than the findings of the present study.

Our investigation found that, among the workers, 8.2% had healthy gingiva, 13.8% exhibited bleeding, 31.8% had calculus, 28.2% had shallow pockets, and 20.4% had deep pockets while a study conducted by Singh et al. [8] on factory workers found 0% had healthy gingiva, 4.45% had bleeding problem, 80.17% had calculus, 7.79% had shallow pockets, and 0.8% had deep pockets.

In this present study, 91.5% workers had gingival and periodontal problems, which were similar to marble mining workers of Udaipur city (i.e., 96.5%) [9].

The result of this present study was also similar to the result of a study done by Pilot et al. [10]; 97% of the Finnish industrial workers had gingival and periodontal problems.

In the present study, 20.4% workers had deep pockets of more than 6 mm, while a study conducted by Solanki et al. [11] in the marble stone mining workers of the Jodhpur city reported of 33.4% deep pockets.

The present study also found that periodontal health of footwear workers was better than that of the tanning unit workers. This was comparable to the study performed by Lie et al. [4] among aluminum factory workers where it was found that the administrative unit employees had better periodontal health than the factory workers.

While only 8.2% had healthy gingiva, 42.8% and 48.9% had gingival disease and periodontal disease, respectively. Healthy periodontium and bleeding were seen only in younger age groups (20–35 years), while the prevalence of deep pockets increased with age. A study conducted by Vanishree et al. [12] among female beedi factory workers found that only 0.47% had healthy periodontium and 16.26% and 83.25% had gingival disease and periodontal disease, respectively, which are less as compared to those of the present study.

The Community Periodontal Index score 2 was found more in male (36.1%) than in female (25.8%); this was less than the result of study conducted by Bansal and Veeresha [13], which found CPI score 2 to be 58.4% in males and 48.8% in females.

The present study also found that prevalence of periodontal disease is directly proportional to age which is comparable to the result of the studies conducted by Dini and Guimaraes [14], Miyazaki et al. [15], van Palenstein Helderman et al. [16], Krustrup and Erik Petersen [17], Weirzbicka et al. [18], and Davies et al. [19].

The mean number of sextants with calculus was 2.05 ± 2.11; this was lower than the finding of a study conducted by Vanishree et al. [12] who found the mean number of sextant to be 4.87 ± 2.12 among beedi factory workers.

The prevalence of periodontal disease among males (90.6%) was lower than that among females (91.5%); this was comparable to the findings of a study conducted by Sundhansu et al. [20] who found the prevalence rate among females to be 96.4%.

Calculus was found to be most widespread among the 39–48 years age group (63.1%), whereas the prevalence of shallow periodontal pockets was higher in the 29–38 years age group (69.4%); this was in conformation with the finding of the study conducted by Lie et al. [4] among aluminum factory workers.

The prevalence of the periodontal pocket was highest among the 20–35 years ages group. This was in contrast to the findings of the study performed by Mishra et al. [21] who found that the 31–40 year age group had the highest prevalence of periodontal pocket.

The present study also found that 31.8% of the workers had calculus, 11.1% gingival bleeding, and 28.2% a periodontal pocket of 4-5 mm. Solanki et al. [11] in their study found that 39.6% workers had calculus and 10% had a periodontal pocket of 4-5 mm.

A study conducted by Dagli et al. [22] and Bali et al. [23] also found that the rate of periodontal disease was 85.6% and 98%, respectively.

Among 850 workers, loss of attachment (LOA) 0 code was found among 351 (41.3%) workers: code 1 was in 194 (22.8%), code 2 in 96 (11.3%), code 3 in 48 (5.6%), and code 4 in 161 (18.9%) workers. LOA was found to be more in tanning plant workers (136 (21.8%)) than in footwear plant workers (25 (11.1%)). Loss of attachment was found to be more in males than in females. A study conducted by Singh et al. [8] found loss of attachment 0 code among 55.01% workers: code 1 was in 35.6% workers, code 2 in 5.2%, code 3 in 0%, code 4 in 0%, and code *X* in 4.27%. It was also seen that that loss of attachment was found more in workers as compared to administrative staff.

The periodontal health of factory workers globally, as described in the majority of surveys, is generally poor. Specific occupational environmental factors may be a contributing factor to the incidence of periodontal disease. Following are the key factors associated with the high incidence of periodontal disease among factory workers: a high rate of tobacco consumption, poor oral hygiene, poor nutrition, and high occupational stress.

## 5. Conclusion

The findings of this study provide an overview of oral health and periodontal status of leather factory workers. A comprehensive understanding of the extent of the public health problem would enable an effective planning of intervention measures. A health promotion program is highly desirable in this study population which addresses the need of importance of maintaining oral hygiene, habit counseling, and regular visits to a dentist.

## Figures and Tables

**Table 1:** Demographic distribution of factory workers according to age group, gender, and plant.

	Plant		Total	*χ* ^2^ value	*p* value
	Tanning	Footwear			
*Age group (in years)*					
20–35	198 (35.7%)	192 (48.4%)	390 (45.8%)	5.56	0.06
36–50	146 (26.3%)	134 (33.8%)	280 (32.9%)		
>50	110 (19.8%)	70 (17.6%)	180 (21.1%)		

*Gender*					
Male	262 (52.6%)	236 (47.3%)	498 (58.5%)	0.3	0.5
Female	192 (54.5%)	160 (45.4%)	352 (41.4%)		
Total	454	396	850		

**Table 2:** Distribution of the periodontal status among subjects according to age group, gender, and plant.

		Healthy 0	Bleeding 1	Calculus 2	Shallow pocket 3	Deep pocket 4	*χ* ^2^ value	*p* value
*Age groups (year)*	20–35	40 (10.2%)	30 (7.6%)	136 (34.8%)	112 (28.7%)	72 (18.4%)	71.0	0.001 (HS)
	36–50	20 (7.1%)	40 (14.2%)	98 (35%)	91 (32.5%)	31 (11.07%)		
	>50	10 (5%)	25 (13.8%)	37 (20.5%)	37 (20.5%)	71 (39.4%)		

*Plant*	Tanning	20 (4.4%)	52 (11.4%)	113 (24.8%)	137 (30.1%)	132 (29%)	68.9	0.001 (HS)
	Footwear	50 (12.6%)	43 (10.8%)	158 (39.8%)	103 (26%)	42 (10.6%)		

*Gender*	Male	40 (8%)	36 (7.2%)	180 (36.1%)	110 (22.2%)	132 (26.1%)	61.2	0.001 (HS)
	Female	30 (8.5%)	59 (17.7%)	91 (25.8%)	130 (36.9%)	42 (11.9%)		
	Total	70 (8.2%)	95 (11.1%)	271 (31.8%)	240 (28.2%)	174 (20.4%)		

HS, highly significant.

**Table 3:** Distribution of the mean number of sextant affected by periodontal disease according to age group, gender, and plant.

		Healthy 0	Bleeding 1	Calculus 2	Shallow pocket 3	Deep pocket 4
		Mean ± SD	Mean ± SD	Mean ± SD	Mean ± SD	Mean ± SD
*Age groups (year)*	20–35	1.82 ± 0.69	0.88 ± 1.42	1.25 ± 1.51	1.33 ± 1.54	0.43 ± 1.32
	36–50	0.37 ± 0.78	1.86 ± 1.33	1.68 ± 1.655	1.55 ± 1.43	0.73 ± 1.59
	>50	0.12 ± 0.48	2.29 ± 1.76	2.05 ± 2.11	1.89 ± 1.80	2.37 ± 2.33
	Kruskal-Wallis test	**9.48**	**7.81**	**13.27**	**16.26**	**18.46**
	*p* value	**0.15**	**0.32**	**0.01**	**0.001 (HS)**	**0.001 (HS)**

*Plant*	Tanning	0.91 ± 072	1.1 ± 1.63	1.2 ± 1.70	1.2 ± 1.59	1.34 ± 2.06
	Footwear	1.3 ± 0.37	2.1 ± 1.48	1.3 ± 2.40	1.6 ± 1.61	1.2 ± 0.824
	Mann–Whitney *U* test	**211.19**	**51.34**	**317.16**	**15.17**	**269.52**
	*p* value	**0.001 (HS)**	**0.32**	**0.001 (HS)**	**0.42**	**0.001 (HS)**

*Gender*	Male	0.8 ± 0.79	1.4 ± 1.62	1.2 ± 1.50	1.3 ± 1.56	1.6 ± 2.07
	Female	1.5 ± 0.29	1.8 ± 1.57	1.7 ± 2.38	1..5 ± 1.63	0.9 ± 1.411
	Mann–Whitney *U* test	**157.91**	**0.49**	**189.53**	**12.47**	**323.19**
	*p* value	**0.001 (HS)**	**0.27**	**0.001 (HS)**	**0.48**	**0.001 (HS)**

**Table 4:** Distribution of the loss of attachment among factory workers according to age group, gender, and plant.

		0–3 mm code 0	4-5 mm code 1	6–8 mm code 2	9–11 mm code 3	>12 mm code 4	*χ* ^2^ value	*p* value
*Age groups (year)*	20–35	202 (51.8%)	68 (17.4%)	48 (12.3%)	8 (2.1%)	64 (16.4%)	192.0	0.001 (HS)
	36–50	115 (41.1%)	101 (36.1%)	33 (11.8%)	8 (2.9%)	23 (8.2%)		
	>50	34 (18.9%)	25 (13.9%)	15 (8.3%)	32 (17.8%)	74 (41.1%)		

*Plant*	Tanning	187 (41.1%)	122 (26.8%)	42 (9.2%)	17 (3.7%)	86 (18.9%)	16.8	0.002 (HS)
	Footwear	164 (41.4%)	72 (18.1%)	54 (13.6%)	31 (7.8%)	75 (18.9%)		

*Gender*	Male	207 (41.6%)	87 (17.5%)	52 (10.4%)	40 (8%)	112 (22.5%)	36.0	0.001 (HS)
	Female	144 (40.9%)	107 (30.4%)	44 (12.5%)	8 (2.3%)	49 (13.9%)		
	Total	351 (41.3%)	194 (22.8%)	96 (11.3%)	48 (5.6%)	161 (18.9%)		

**Table 5:** Distribution of mean number of sextant by loss of attachment of subjects according to age code, gender, and plant.

		0–3 mm (0)	4-5 mm (1)	6–8 mm (2)	9–11 mm (3)	>12 mm (4)
		Mean ± SD	Mean ± SD	Mean ± SD	Mean ± SD	Mean ± SD
*Age groups (year)*	20–35	3.99 ± 2.36	0.7 ± 1.13	0.40 ± 0.80	0.22 ± 0.64	0.17 ± 0.60
	36–50	3.78 ± 2.07	0.70 ± 1.05	0.40 ± 0.80	0.37 ± 0.79	0.52 ± 1.24
	>50	2.22 ± 2.28	1.41 ± 1.51	0.44 ± 0.83	1.33 ± 1.17	1.28 ± 1.61
	Kruskal–Wallis test	**35.89**	**11.5**	**5.6**	**21.89**	**28.32**
	*p* value	**0.001 (HS)**	**0.6**	**0.85**	**0.001 (HS)**	**0.001 (HS)**

*Plant*	Tanning	3.43 ± 2.42	0.86 ± 1.31	0.41 ± 0.81	0.61 ± 1.00	0.66 ± 1.31
	Footwear	3.87 ± 2.11	1.11 ± 1.31	0.40 ± 0.80	0.28 ± 0.71	0.31 ± 0.95
	Mann–Whitney *U* test	**78.89**	**5.21**	**19.8**	**241.49**	**385.93**
	*p* value	**0.001 (HS)**	**0.54**	**0.7**	**0.001 (HS)**	**0.001 (HS)**

*Gender*	Male	3.44 ± 2.44	0.80 ± 1.26	0.39 ± 0.79	0.66 ± 1.02	0.69 ± 1.34
	Female	3.70 ± 2.21	1.12 ± 1.36	0.43 ± 0.82	0.34 ± 0.78	0.39 ± 1.04
	Mann–Whitney *U* test	**85.31**	**4.97**	**12.8**	**347.13**	**389.48**
	*p* value	**0.001 (HS)**	**0.59**	**0.16**	**0.001 (HS)**	**0.001 (HS)**

HS = highly significant.

## Data Availability

Data were collected through modified WHO-1997 Oral Health Assessment Performa and analyzed through SPSS. The results of this study are included within the article.

## Additional Points

*Recommendations*. (1) Regular health checkup for the workers at their workplace is advised. (2) Provision of basic required protective devices and clothing to prevent them from chemical and environmental hazards. (3) Improving the needed facilities at work place. (4) Health education and appropriate counseling should be given to the health workers regarding the illness that can occur commonly in leather industries. (5) Through the Ministry of Labor and Employment, Government of India and Labor Department of State and Union Territories, new health programs should be initialized.
